# Microwave Devices for Wearable Sensors and IoT

**DOI:** 10.3390/s23094356

**Published:** 2023-04-28

**Authors:** Alessandra Costanzo, Elisa Augello, Giulia Battistini, Francesca Benassi, Diego Masotti, Giacomo Paolini

**Affiliations:** Department of Electrical, Electronic, and Information Engineering (DEI) “G. Marconi”, Alma Mater Studiorum—University of Bologna, 40136 Bologna, Italy; elisa.augello2@unibo.it (E.A.); giulia.battistini13@unibo.it (G.B.); francesca.benassi9@unibo.it (F.B.); diego.masotti@unibo.it (D.M.); giacomo.paolini4@unibo.it (G.P.)

**Keywords:** wearable, electronics, sensors, IoT, e-Health, microfluidics, localization, fall detection, 3D printing, energy harvesting, wireless power transfer

## Abstract

The Internet of Things (IoT) paradigm is currently highly demanded in multiple scenarios and in particular plays an important role in solving medical-related challenges. RF and microwave technologies, coupled with wireless energy transfer, are interesting candidates because of their inherent contactless spectrometric capabilities and for the wireless transmission of sensing data. This article reviews some recent achievements in the field of wearable sensors, highlighting the benefits that these solutions introduce in operative contexts, such as indoor localization and microwave sensing. Wireless power transfer is an essential requirement to be fulfilled to allow these sensors to be not only wearable but also compact and lightweight while avoiding bulky batteries. Flexible materials and 3D printing polymers, as well as daily garments, are widely exploited within the presented solutions, allowing comfort and wearability without renouncing the robustness and reliability of the built-in wearable sensor.

## 1. Introduction

The advancement of technology bringing connectivity and intelligence closer to users is facilitating the development of next-generation Internet of Things/Internet of Everything (IoT/IoE). The escalation of compact and affordable computing devices that allow for the massive distribution of noninvasive interfaces, which bring real-time sensing capabilities, as well as wireless communication properties, enables the concept of “wearable computing” [1]. Nevertheless, the market shows an increasing trend in demand for wearable solutions, which have gained widespread popularity in various fields, including medicine, security, entertainment, and industrial [2]. Wearable devices are a new class of user-centric technology available in everyday life that enable individuals to gather and analyze data in real time, based on their personal activities and behaviors. Their incorporation into everyday objects has the potential to personalize data collection and interaction with the surrounding environment at the user level. These devices typically consist of a combination of sensors, antennas, and a variety of other electronic components properly designed to be worn on the body.

The biomedical field, as well as the e-Health scenario, are key areas where wearable devices are being widely used, showing their great potential to revolutionize the way we monitor our health and well-being daily, owing to their ability to provide continuous and instantaneous information of physiological data [3,4]. Wearable technology allows for the monitoring of a wide range of biometric data, including heart rate, respiratory rate, blood pressure, glucose levels, and so on [5,6,7,8,9]. However, their integration into clothing to maximize comfort is of fundamental importance to avoid interference with the user’s activities and to have accurate retrieval of data. Diffusion of on-body technology is useful not only for patients but also for healthcare providers who can benefit from remote monitoring of vital signs. On a large scale, there is the possibility of revolutionizing the clinical research approach to collect valuable data on various medical conditions, identifying patterns in a more efficient way with respect to traditional clinical trials.

The specific requirements for wearable devices vary depending on their intended application, but the most common ones are as follows:

- Energy autonomy to have reliable and continuous functioning;
- High level of miniaturization and seamless structure to reduce as much as possible the device’s intrusiveness and to reach a high level of integration;
- Sensing and localization capabilities to provide large amounts of information about the object or the environment being sensed;
- Reliability in data acquisition, particularly in the biomedical field, to avoid incorrect diagnoses and treatments.

The successful diffusion of wearable devices for everyday use is closely related to their ability to remain powered and operational over an extended period. The bottleneck of wearable electronics is their battery duration, which limits their reliability and lifetime. Furthermore, enabling functioning of the device that is independent from traditional power sources, such as battery-powered or wired solutions, can reduce maintenance and interventions required related to battery replacement. Therefore, research is progressing toward the goal of removing batteries partially or completely from wearable electronics and devices. Supported by these motivations, energy harvesting (EH) and wireless power transfer (WPT) technologies place themselves as promising solutions to wirelessly energize wearable devices and sensors in an efficient way. Novel advancements in microelectronics and low-power microsensors allow for a noticeable reduction in power consumption, promoting the employment of EH and WPT by adding energy management circuits and energy storage elements to the device while maintaining its reduced dimensions [10]. The energy autonomy required by wearable systems, which can be either based on active devices or passive ones, can be reached by exploiting intentional radiofrequency (RF) sources or through energy scavenging. For the latter, a variety of energy sources present in the environment are frequently exploited and converted into useful power to energize wearable devices [11]; EH technologies include solar and thermal energy, as well as piezoelectric-based devices [12]. In addition, human-powered energy harvesting is another solution, exploiting biochemical sources from chemical reactions or biomechanical energy generated through human body movement [13]. Energy harvesting combined with on-demand power transmission will pave the way for the reliable and sustainable exploitation of wearable devices in the healthcare field.

**Figure 1:**
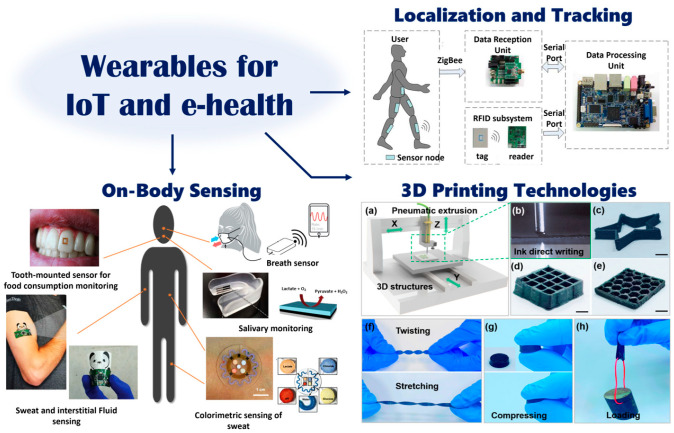
Envisioned scenario of an integrated system for wearable sensing [14], indoor localization [15], and (**a**–**h**) 3D printing techniques [16] for e-Health applications.

In this context, the efficient supply of energy to wearable devices allows for the investigation of their usage in multiple application fields, as shown in Figure 1. This illustration schematically represents the main potential applications where wearable antennas can be exploited, namely communication, sensing, and localization. These are indeed the three different aspects touched on in this review that are related to microwave wearable IoT devices for on-body sensing, localization, tracking, fall detection, and 3D printing techniques for the realization of flexible sensors that could be easily worn by users.

Wearable antenna sensors exploit different frequencies, such as microwave or RF, and it is possible to classify them into different categories based on the information provided. Operations such as dielectric, strain, temperature, and crack sensing [17] can be extensively found in the literature. They detect a variety of information from different application fields, spacing from manufacturing to quality monitoring. Usually, the functioning principle of antenna sensors relies on the interaction between electromagnetic (EM) waves and dielectric properties, since these are engineered to have exceptional sensitivity with respect to variations in the permittivity of the material considered.

Focusing on the healthcare field, the dielectrics under investigation correspond to biological tissues; therefore, wearable sensors aim to convert biological reactions into quantifiable signals. Any changes in the permittivity spectrum and level correspond to an irregularity or a change in the concentration of specified molecules, providing monitoring and, eventually, an alert of health issues. EM wave sensing has found a variety of applications in the medical domain, including the detection of glucose [18], cancer cells [19], and skin hydration levels [20], as well as cardiac monitoring [21] and other biological data [22].

Another application field examined in this paper regards the localization capability of wearable devices. Positioning capabilities are highly required and of fundamental importance to enable the concept of the Smart Hospital [23]. This technology indeed makes it extremely effective in meeting the increasing demand for home-based hospitalizations, particularly when it comes to patients requiring long-term care. Real-time monitoring of location is crucial to identify the position of the person within indoor environments and to have a prompt reaction when an emergency detection happens [24]. There are multiple challenges in effectively realizing these kinds of sensing devices since they usually require proper coordination among wearable and ambient sensors, keeping the system low-cost and nonintrusive to be used on a large scale. Multiple prototypes are present in the literature, highlighting the importance of wearable sensors when it comes to elderly monitoring. For instance, posture recognition combined with indoor localization allows the device to provide alerts when the patient lies down in an unusual place [15]. Unintentional falls are one of the main causes of injuries in the elderly population, so fall detection and fall prevention are hot topics in the world of e-Health. An extensively researched technique is to utilize monitoring cameras to identify falls [25]; unfortunately, successful detection is strictly dependent on the camera’s range of visibility, incurring expensive costs when it must be expanded. In some cases, the systems are based on accelerometers [26,27] or radio frequency identification (RFID) tags [28,29,30], validating the use of wearable sensors in fall detection systems. Wearable technology, thanks to its portability, compactness, and versatility in adapting to different parts of the body, is a promising and affordable solution in the healthcare setting.

The integration of wearable systems into everyday life requires a noninvasive structure, leading to a design procedure focused on their miniaturization to have a low profile and be lightweight. The appealing qualities of wearable antennas and sensors, including their portability, low weight, flexibility, and low cost, have gained significant attention in recent years [14,17]. In the literature, it is possible to find a variety of wearable devices [31,32,33] exploring different technologies and antenna structures with the aim of reaching proper levels of efficiency while keeping a structure that is comfortable to wear. Additionally, these devices must be capable of operating in proximity to the human body, providing minimal degradation due to body coupling and dispersion from biological tissues. These requirements make the design of wearable antennas and sensors a cumbersome task, particularly when considering their susceptibility to structural deformation and their challenging fabrication. The materials involved must tolerate bending, contraction, and other mechanical deformations, making it necessary to rely on advancements in available materials and fabrication techniques. This encouraged a deep investigation to find innovative substrates with physical, thermal, and mechanical properties suitable and stable for on-body applications [34]. Traditional fabrication techniques, such as mechanical machining and micromachining, result in devices that are old-fashioned and not suitable when it comes to the generation of some structures. The materials involved in wearable substrates typically require complex shapes and intricate internal structures to reach some predefined electromagnetic properties [17]. Within this framework, the utilization of three-dimensional (3D) printing technology for dielectric materials allows manufacturers to have a high level of precision in fabrication and to create multi-material objects in a single process, employing various materials, such as ceramics, plastics, polymers, nylons, etc. This technology builds the material shape layer by layer, eliminating the need for machining, avoiding material waste, and providing a straightforward procedure to fabricate complex structures.

The main goal of this review paper is to analyze some of the multiple aspects that are included in the wide field of microwave wearable devices. In particular, the authors focused their attention on three aspects they believe have certain experience and can therefore be evaluated in depth. In particular, the paper is organized into three distinct sections, each addressing in detail different aspects of these three topics to provide an overview of innovative technologies and applications of wearable devices for implementation in the e-Health paradigm. Section 2 examines microwave wearable sensing, focusing on microfluidic systems. Section 3 focuses on wearables for indoor localization technologies and fall detection. Section 4 provides an overview of 3D printing technologies.

## 2. Microwave Wearable Sensing

The exploitation of RF and microwaves to design passive and wearable sensors has acquired remarkable interest in recent decades thanks to the many advantages and benefits this technology involves, such as the possibility of accessing continuous monitoring of physiological parameters [35,36,37,38], drug delivery [39], or identification of specific substances through biological media [40]. Lightness, compactness, and miniaturization, together with reliability, are key points to be addressed when designing new and advanced microwave wearable sensing systems.

Indeed, the majority of these passive RF or microwave-activated microfluidic wearable sensors are based on dielectric spectroscopy, which has been found to be a label-free and noninvasive technique for analyzing biological substances for different purposes (from fluid identification to cancer cell detection), as extensively described in [41].

Most systems embed microfluidic channels [42] to perform accurate sensing of biological or nonbiological fluids that are present on the body surface in order to perform further analysis, achieving the realization of so-called lab-on-chip technology [43]. The fundamental operating principles are based on the interaction between microwaves and biological matter and the relationship between variations in dielectric permeability and the permittivity of the different types of tissues or cells that are to be analyzed and characterized. Such an approach has a wide range of applications and could be a game changer in the early detection of severe diseases [44]. The need to find alternative methods to replace physical or chemical transduction has led to the identification of microwave dielectric spectroscopy as a viable candidate, particularly in situations in which the use of biological markers is not required. This implies a significant reduction in the use of chemical products as reagents and solvents, as well as a notable decrease in operational time. Microwave transduction is regarded as a valid method for analyzing not only biological liquids but also smaller molecules up to the cell level and ensuring rapid and nondestructive biological characterization. The monitoring of sweat biomarkers is one of the most noninvasive and easily accessible solutions for performing real-time wearable analysis of important physiological parameters. In this context, in order to achieve wearability and detection efficiency, the exploitation of RFID technology, optimized to embed microfluidic channels, can be a suitable solution. In [45], a wearable, adhesive RFID-based sensor was designed to perform chronological monitoring of specific biomarkers contained in sweat that was made to conform to the shape of the human body. A commercial RFID chip was adapted with few components to allow potentiometric sensing of electrolytes in sweat, as well as skin surface temperature, which could be useful for hydration and heat-stress monitoring. The patch is battery-free, and the readout is enabled wirelessly by means of a smartphone equipped with a dedicated app that uses near-field communication (NFC) technologies, working at a frequency of 13.56 MHz.

The system was derived on a thin flexible substrate, the Dupont Pyralux AC, which has an 18 μm thick copper foil cladded by 12 μm of Kapton. The electrodes were fabricated by electrodeposition and then connected to the chip circuitry to enable the readout. The RFID chip was integrated and connected to the antenna coil after validating the correct impedance matching. The microfluidic paper layer was interposed between the skin and the circuitry and allowed sweat to be directed to the sensing area. The readout was performed by analyzing the concentration of sodium (Na^+^) when tested within a range of variation between 20 and 70 mM, showing a dynamic range of 235 ÷ 255 mV, which achieved 96% accuracy of the correct Na^+^ concentration when a 0.5 W RF power was transferred from the reader [45]. A linear response of the system, in terms of the value of potential measured by the electrode, is expected when the Na^+^ concentration varies from 20 to 70 mW, resulting in values in the range from 176 to 200 mV.

**Figure 2:**
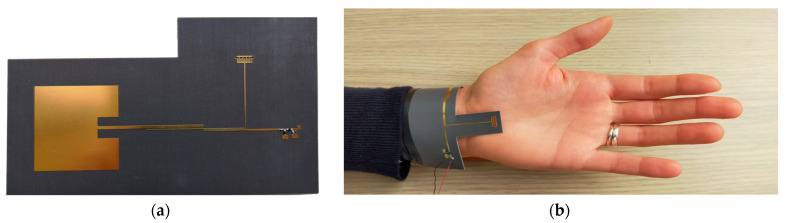
(**a**) Picture of the prototype, showing the narrowband antenna for power reception, the coupled-line filter loaded with the microfluidic stub resonator, and the rectifier circuit; (**b**) picture of the system worn as a bracelet [46]. © 2021 IEEE.

In the field of wearable sensing, there are several molecules that can be detected, from biomarkers to human cells up to non-biological liquids that can be present on the body surface. In [46], a detector was designed to be used in a medical setting to detect specific solutions, particularly ethanol-based solutions, which are the main components of common hand sanitizer. The previously mentioned operating principles can also be used in reverse, in which the main goal is not to investigate the effect of EM waves on the dielectric properties of the fluid but to exploit the effect of its presence within a resonant structure as a material causing a variation in the properties of the hosting structure, resulting in a shift in its resonant frequency. The sensor was intended to detect the presence of an ethanol solution on the body surface using a meandered microfluidic channel excavated on an RT/Duroid 5880 bendable base. The fluids in the channel are used as media inserted between the metal fingers of an interdigitated capacitor, meandered in such a way that the sensing area is increased while the volume is kept comparatively small. The channel is placed on top of a stub that is adjusted to resonate as an open circuit at 2.45 GHz when the channel is filled with an ethanol solution (70% concentration), which is easily found in standard hand sanitizers. When the channel is filled with water or kept empty, the resonant frequency of the stub input impedance changes completely. The resonator is placed in substitution of one open end of a second-order coupled-line filter to wirelessly power the device and carry out fluid sensing. Frequency discrimination is guaranteed by the loaded filter, and its efficacy is solely dependent on the channel’s material. A narrowband antenna is connected to the input port of the coupled-line filter, and a full-wave rectifier is connected to the output port. The coupled-line filter also acts as the matching network between the antenna and the rectifier, in addition to achieving frequency discrimination. The realized prototype is shown in Figure 2.

**Figure 3:**
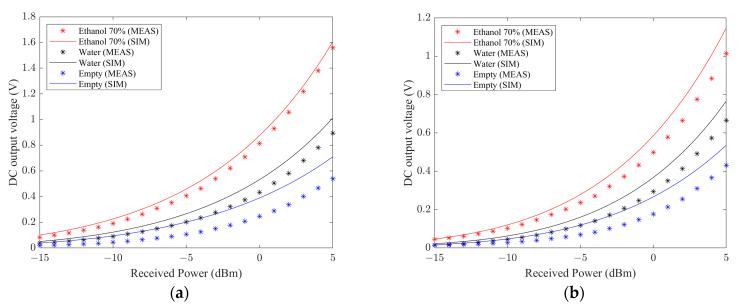
DC output voltage level vs. received power for (**a**) open circuit conditions and (**b**) adopting an optimized load of 6.5 kΩ [46]. © 2021 IEEE.

To accurately read the proper detection of the ethanol solution, the sensor was optimized using harmonic balance (HB) simulations with the aim of optimizing the radiofrequency-to-direct-current (RF-to-DC) power conversion efficiency and the direct current (DC) output voltage. The obtained results, displayed in Figure 3, show the DC output level for different received RF powers, showing the robustness of the system in the whole power range in terms of detection and differentiation of the searched solutions with respect to water and with respect to the total absence of solution in the channel and thus on the body surface.

Thanks to the advancing progress in additive manufacturing, microfluidic sensors have undergone an expanding development that has paved the way for miniaturized but highly accurate designs. In [47], a flexible RF sensor, embedding microfluidic channels and realized by means of additive manufacturing, was developed that enabled the accomplishment of a highly sensitive fluid sensor to be placed on the body surface. The reusable flexible sensor was designed to differentiate several fluids, such as hexanol, glycerol, ethanol, and water. A dual-spiral slot resonator inserted into two ground planes of a coplanar waveguide (CPW) was part of the structure of the realized prototype.

When permittivity values change in the slot, they significantly alter its capacitance and cause a shift in its resonant frequency; thus, the slot structures are ideally suited for microfluidics-based sensing. This makes the resonant shift an RF parameter that might be used for the wireless sensing of permittivity changes.

The strength of this design is the correlation between the shift in the resonant frequency of the insertion loss and the fluid present in the microfluidic channel. For testing purposes, consequent drops of seven different fluids were used, and the scattering parameters of the two-port system were evaluated. The measurements were performed considering different fluids that were being tested. First, single-fluid solutions were considered, experiencing a resulting insertion loss whose resonant frequencies varied from a minimum of 2.1 GHz when the channel was filled with water to 3.9 GHz when the channel was left empty. Subsequently, some water–glycerol solutions of different concentrations were considered, showing a frequency shift range spanning from 2.1 to 3.5 GHz. The overall maximum shift within the measurement frequency band was 43.8% [47], which happened when passing from an empty channel to a channel filled with water, due to the drastically different relative permittivity values (from ε_r_ = 1 for the empty channel to ε_r_ = 73 when water was present). The presented reusable sensor, called “peel-and-replace”, boasts promising performance, paving the way for the development of microwave-based wearable sensors to be used for the monitoring of several substances, leading to the design of future “smart-skin”.

**Figure 4:**
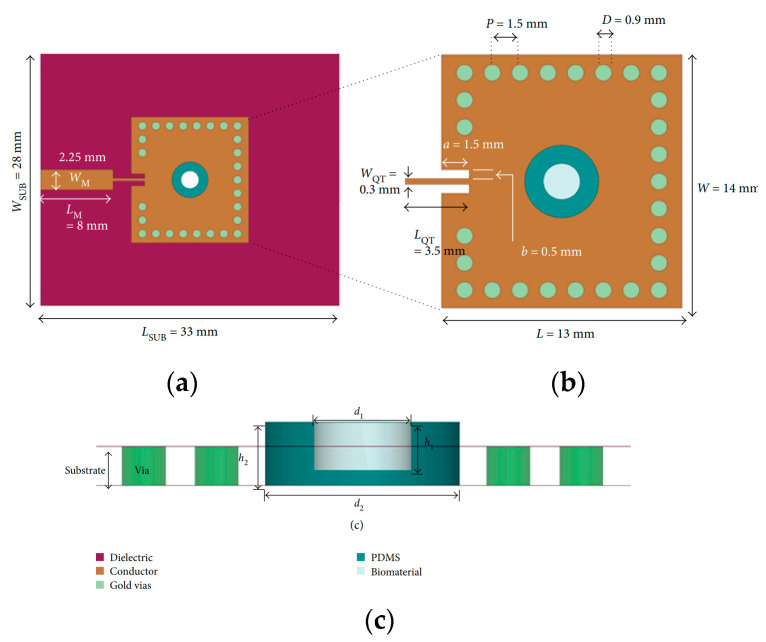
(**a**) Front, (**b**) back view, and (**c**) stack-up of the microfluidic biosensor [49].

In the medical field, microfluidic sensors can be exploited to detect biomolecules up to the cellular level [48]. Due to the reduced dimensions of the molecules to be analyzed, these types of microfluidic sensors must function at higher frequencies to ensure the accuracy and sensitivity of the detection. In [49], a substrate-integrated waveguide (SIW) cavity resonator was used to build a microfluidic biosensor. The suggested sensor used the frequency shift in the microfluidically loaded SIW cavity to identify fibroblast cells. The noninvasiveness of the proposed system was one of the main characteristics of the design, together with compactness, low cost, and quick fabrication procedure. The main components of the systems are the patch antenna and the polydimethylsiloxane(PDMS)-based cup-shaped microwell. A schematic representation is shown in Figure 4.

Fluid detection was performed by analyzing the shift in the resonant frequency of the resonator due to the dielectric perturbation induced by the presence of the fluid being tested. To achieve this, a microwell was located where the electric field magnitude experienced the maximum, which corresponded to the center of the SIW cavity. Different diameters and thicknesses of the PDMS and the microwell were simulated to investigate the detection performance, which was seen as a shift in the resonator resonant frequency. The optimized value for the PDMS diameter was found to be 4 mm.

**Figure 5:**
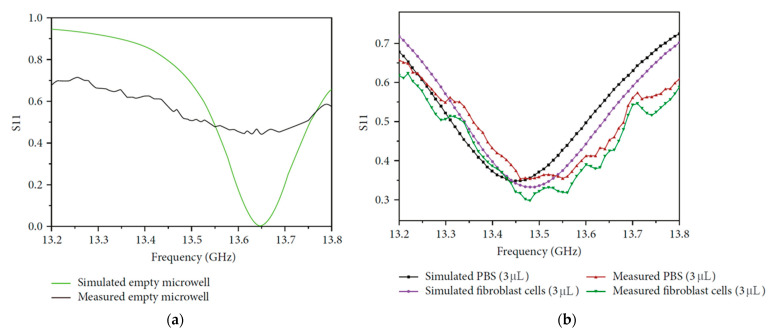
Simulated and measured reflection coefficient in the case of (**a**) empty channel and (**b**) PBS solution and in the presence of fibroblast cells [49].

The biosensor was subsequently tested with different fluids filling the microwell, and the sensitivity was computed, accounting for the resonant frequencies obtained when the patch was not loaded and when the microwell was filled with phosphate-buffered saline (PBS) solution. Subsequently, the fibroblast cells were located in the microwell, and the overall shift in the resonant frequency was measured to be 170 MHz with respect to the condition of an empty channel, as shown in Figure 5, thus allowing for the safe identification of tested cells with only 3 μL of fluid required.

In this last part of the section, three works have been presented, with the aim of describing three different systems for detecting three different materials: in the first case, sweat [45]; in the second case, ethanol solutions [46]; and in the third case, fibroblast cells, outlining the versatility that these systems may boast in the field of biological and nonbiological fluid identification in the wearable context [49].

## 3. Wearable RFID Sensors for Indoor Localization Systems and Fall Detection

### 3.1. Indoor Positioning System

The field of RFID and wireless sensor networks (WSNs) has expanded significantly in recent years, and the current trend is toward the integration of cutting-edge wireless technologies into areas of daily life to transform these into Smart Spaces that include all pertinent IoT technologies. With its capacity to remotely identify and distinguish goods and persons, even in crowded surroundings and electromagnetically harsh environments, in retirement communities or private homes, RFID is especially crucial. The fact that the average age of the world’s population is significantly rising has an impact on the number of elderly people. As a result, it is becoming more and more important to monitor elderly people’s movements and their behaviors in order to identify any age-related illnesses or problems as soon as possible (e.g., Alzheimer’s disease and senile dementia). This section demonstrates how these technologies can be effectively used to localize people in their daily lives by observing them and nonintrusively assessing their routines.

One of the newest and most intriguing technologies is the indoor positioning system (IPS), and there are many ways to activate it. For example, in recent years, many solutions based on RFID have enabled indoor localization [50,51], detection of movements of different human body segments [52], and posture recognition [23,53], as well as modules exploiting the reception of ultrawideband signals (UWB) and information coming from inertial measurement unit (IMU) sensors [54].

**Figure 6:**
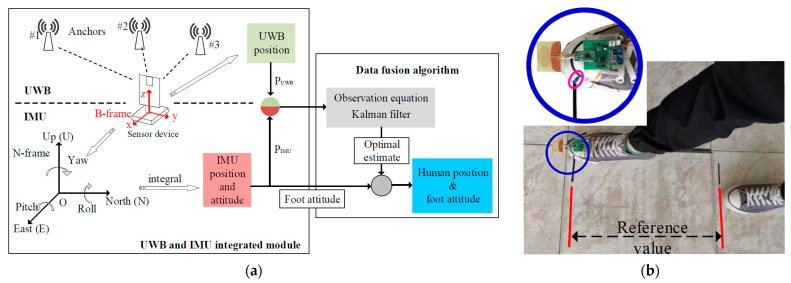
(**a**) Schematic view of the operation principles of a module integrating UWB and IMU technologies. #1, #2, #3 represent the three UWB anchors distributed in the environment. (**b**) Circuitry and UWB antenna worn by the user in the foot. The pink circle highlights the marker point on the user’s shoe adopted during the experiment [54].

As can be seen in Figure 6, this last technology previews the employment of several UWB receivers (or anchors) widely distributed inside the room being tested, as well as a significant amount of time for the analysis of the incoming data. The main advantage is that it allows the avoidance of the potential effects of fading and shadowing that may occur in indoor environments at certain frequencies [55,56].

**Figure 7:**
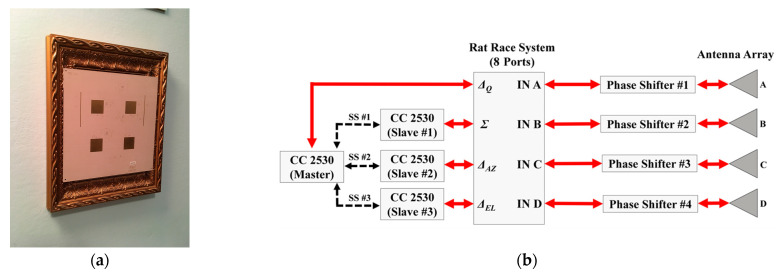
(**a**) Photograph of the RFID reader included in a frame; (**b**) block diagram of the RF and digital circuitry of the reader for electronic tridimensional beam scanning. The red arrows represent RF signals, whereas the black ones are the digital connections [57]. © 2019 IEEE.

In [57], the tridimensional localization of tagged people in electromagnetically challenging indoor environments is accomplished with a customized 2.45 GHz RFID reader (Figure 7) that makes use of the monopulse radar concept applied to bidimensional electronic beam steering techniques.

The key element for the overall architecture of the RF system is the combination of four bended rat race hybrid couplers that provide the in-phase and out-of-phase received signals, both in the azimuth and in the elevation planes, and an auxiliary signal (see Σ, Δ_AZ_, Δ_EL_, and Δ_Q_ in Figure 7b).

The four microstrip lines supplying the antennas were connected to the monopulse comparator at the same time (antennas A, B, C, and D in Figure 7b). The selected microcontroller unit (MCU) is from Texas Instruments (TI) CC2530, which enables radio communication for each channel in the industrial, scientific, and medical (ISM) 2.4 GHz band and therefore acts as a transceiver; each of the four abovementioned channels is connected to its own CC2530.

A suitable data processing unit was created and utilized to estimate the distance of the tags from the reader, in addition to the previously shown calculation of the two angular positions, with the goal of analyzing people’s motions and the relative heights of the tags. This procedure was accomplished thanks to the assessment of the received signal strength indicators (RSSI) coming from the reader.

**Figure 8:**
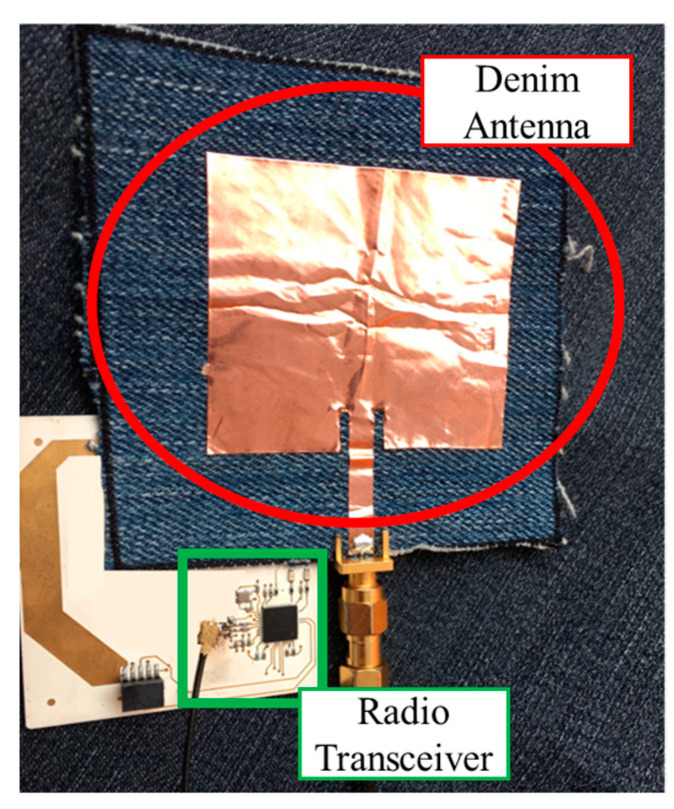
Wearable antenna realized on a denim substrate and connection with the MCU/transceiver circuitry realized on Rogers.

Localization is performed in the most transparent and nonintrusive manner possible. In fact, the development of wearable antenna prototypes on flexible textile materials has been carried out to create a fully worn RFID tag suitable for indoor localization. In particular, the antennas have been replicated on a denim substrate (Figure 8) such that they can be sewn into other common clothing or directly designed into denim apparel [58].

The results of measurements for different setups showed that the average error with wearable tags was 11% for distances up to 4.50 m in a room of 30 m^2^.

### 3.2. Fall Detection Systems

The renovated aim of the previously presented RFID system [57] is also to detect potential falls that could occur to people living alone in indoor environments or in crowded retirement houses. In fact, a mean 3D localization error of 17.6 cm and a mean detection error in height of 8.3 cm were retrieved; the latter suggests the employment of the localization system as a remote fall detector. This research showed that the current microwave system can be a dependable solution to detect falls of elderly people living alone, in communities, or cohousing, thus eliminating the need for ongoing assistance. Indeed, the measurements always led to the correct detection of the fall events, with no false positive or false negative situations. Twelve different fall episodes were tested and verified: lying down face upwards, lying down face downwards, lying down lateral (on both sides), and sitting on the ground, all with reader–tag distances of 0.5, 2, and 3 m. A further advantage of this proposed fall detection sensor is that it can operate alone, in real time, and the fall detection alarm takes less than 15 s to be set off.

In that sense, several research studies have been conducted in order to face the difficult issues caused by falls among the growing elderly population, which is a result that has emerged in the aging global society.

**Figure 9:**
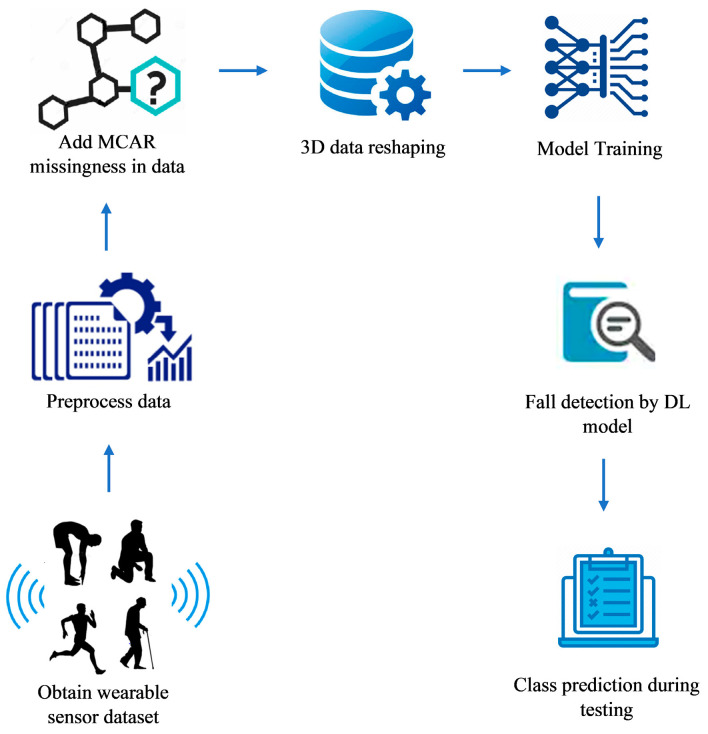
Schematic block diagram representing the principles of operation of a fall detection system based on deep learning algorithms exploiting data coming from wearable sensors [59].

In [59], a noise-tolerant system operating even when the data contained missing values was presented. The system was based on wearable sensors, with the work primarily leveraging deep learning and specifically applying recurrent neural networks with an underlying bidirectional long short-term memory stack. A block diagram of the proposed system is shown in Figure 9. Considering the SisFall and UP-Fall datasets, the system generated an accuracy of 97.21% and 97.41%, a sensitivity of 96.97% and 99.77%, and a specificity of 93.18% and 91.45%, respectively.

An interesting example of a system for fully remote fall detection is presented in [60], where a very noninvasive technology (WiFall) is proposed. It makes use of physical layer channel state information (CSI) to identify human activity in an indoor setting before learning the precise patterns associated with falls and other targeted activities. The three essential components of the WiFall system are sensing, learning, and alerting. The experimental results show that WiFall achieved 90% detection accuracy, with an average false alarm rate of 15%.

## 4. 3D Printing Technologies for Low-Cost Wearable Battery-Free Devices

Emerging 3D printing technologies, or additive manufacturing (AD), are becoming an increasingly attractive option for the manufacturing of RF prototypes in the industrial and academic research fields because of the advanced design capabilities in shaping the bespoke EM properties of the 3D printable material and the cost-efficient, high accuracy, and fast production of RF/microwave components compared to traditional micromachining fabrication processes.

The advantages of AD are not limited to the different possible printing processes (vat photopolymerization, material jetting, material extrusion, and stereolithography, to name a few) but also include the wide variety of materials that can be used, from low-loss dielectric to highly conductive metallic inks and from stiff and rigid materials to flexible and stretchable ones, which allow the extension of the 3D design possibilities of single-material-to-multimaterial additively manufactured circuits and devices, as well as their applications (transmission lines, lenses, metamaterials, filters, or antennas) [61].

The high accuracy resolution of AD technologies, typically in the range of 10 ÷ 200 μm, provides the possibility of designing miniaturized integrated circuits and electronics, which is a key requirement within the framework of portable, wearable, noninvasive, and miniaturized IoT devices for the healthcare monitoring system, which is increasingly moving from being hospital-based to individual-based.

Therefore, the broad acceptance of 3D printing technologies in the design and fabrication of portable wearable microwave electronics and biosensors relies on fast, easy, highly accurate, and low-cost fabrication techniques combined with the 3D ad hoc design freedom of stretchable, flexible, ultra-thin, and lightweight functional materials.

This section summarizes some interesting implementations of 3D printing technologies for the design and fabrication of optimized substrates of wearable microwave rectennas for WPT applications.

Within this framework, in [62], it has been demonstrated that a low-cost, lossy, flexible, 3D printable resin material, such as the Flexible-80A, distributed by Formlabs (Somerville, MA, USA), can be internally engineered by means of the electromagnetic computer-aided design (CAD) simulation software CST (Computer Simulation Technology) Microwave Studio in order to modify its effective relative permittivity and loss tangent, to mitigate the intrinsic losses caused by the propagation within such a lossy dielectric, and to improve the radiation efficiency when used as substrate of a miniaturized 868 MHz rectifying antenna (rectenna), achieving performances comparable to those realized on conventional RF substrates.

Since these innovative 3D printable materials are not conventionally used for RF/microwave applications, it is mandatory to provide a characterization of the material’s EM properties in the frequency range of interest.

**Figure 10:**
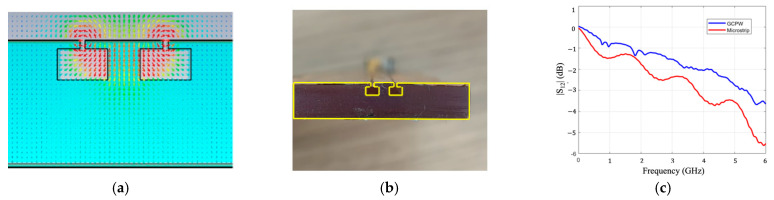
Simulated (**a**) and fabricated (**b**) GCPW on Flexible-80A substrate. (**c**) Comparison between the measured |S_12_| (dB) curves of the microstrip line and a GCPW realized on Flexible-80A [62]. © 2022 IEEE.

In [62], the Flexible-80A, a 3D printable resin material printed using the low force stereolithography (LFS) additive manufacturing process and then post-cured by heating the prototype at 60 °C for ten minutes, gaining a tensile strength of 140%, has been characterized by means of the T-stub resonator method [63] up to 6 GHz, resulting in a relative permittivity ε_r_ = 2.7 and an electrical conductivity σ = 2.34 × 10^−3^·S/m. To minimize propagation losses, Si-micromachining techniques, such as those presented in [64], have led to the idea of selecting CPW technologies as the transmission lines, where the electric field of the quasi-transverse electromagnetic (q-TEM) wave mainly propagates in the air between the microstrip line and the nearby coplanar ground instead of penetrating the lossy dielectric. To further enhance this aspect, it has been verified that removing the dielectric material in the aperture regions (Figure 10a,b) greatly improves the propagation with respect to conventional microstrip lines fabricated on the same lossy substrates. As a demonstration, Figure 10c illustrates a comparison between the transmission coefficients of two transmission lines realized on Flexible-80A, with one being a simple microstrip line and the other being a grounded CPW (GCPW) with air cavities performed in the apertures.

**Figure 11:**
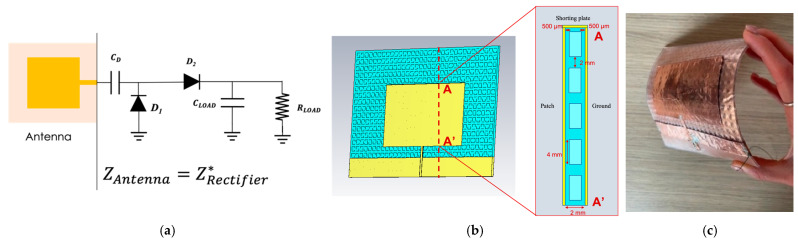
(**a**) Schematic of the 868 MHz rectenna system layout (where Z_Antenna_ is the antenna impedance and Z*_Rectifier_ is the conjugate of the rectifier’s impedance); (**b**) design of the simulated antenna, and (**c**) photograph of the realized prototype of the receiving antenna realized on Flexible-80A with adhesive copper [62]. © 2022 IEEE.

This analysis has led to the design and fabrication of an efficient 868 MHz rectenna on Flexible-80A, whose schematic is shown in Figure 11a, where both the receiving patch antenna and the nonlinear circuit design benefit from the engineering of the 3D-printed substrate. In fact, a wearable receiving antenna was selected as a coplanar-fed patch where, inside the substrate, a pattern of small cubes of material was internally removed, leaving 500 μm thick solid layers for supporting the patch and the ground metallization, as shown in Figure 11. These features led to a radiation efficiency of 15% compared to the 6% of a conventional microstrip antenna realized on a solid Flexible-80A sample. Moreover, to further reduce the intrinsic dielectric losses, the matching network, typically designed between the receiving antenna and the rectifier, was eliminated and thus the patch was designed to show an input impedance conjugate matched to that of the rectifier in correspondence of the operating frequency of 868 MHz and for lower input power levels, where it is more probable the rectenna will be operating.

**Figure 12:**
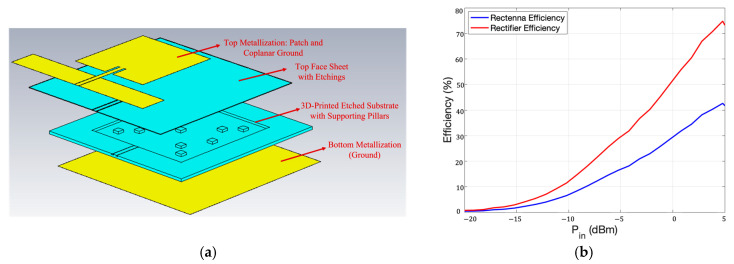
(**a**) Stack-up of the 2.45 GHz rectenna of the receiving antenna with call-out of the transversal cross-section; (**b**) comparison between the measured rectifier and rectenna efficiencies (%) vs. the RF power received by the coplanar-fed patch antenna.

However, since the reduced antenna efficiency degraded the overall effective efficiency of the rectenna, in [65], the substrate was shaped differently with the goal of improving the overall measured rectenna RF-to-DC conversion efficiency. Here, a completely flexible and wearable 2.45 GHz rectenna was realized on Flexible-80A with adhesive copper, based on the results presented in [64]. Thus, the rectenna layout in Figure 11a has been considered with a much better performing wearable coplanar-fed patch as the receiving antenna (stack-up in Figure 12a), where the different substrate design made it possible to reach a measured gain of 6.4 dBi and a simulated radiation efficiency of 57% that is not degraded even when simulated on top of the skin surface. This efficiency enhancement was enabled by the almost complete removal of substrate material from below the patch metallization, inspired by the micromachined patch antennas presented in [66]. Here, the copper layers are supported by thin sheets of solid material and small squared shape pillars, ensuring better mechanical stability to the structure and making sure that the air cavity walls inside the substrate do not touch.

Even in this case, a GCPW with etchings in the apertures and below the microstrip was selected as a feeding transmission line to further reduce the losses intrinsic to propagation. Therefore, the considered design and system layout led to a measured rectenna RF-to-DC efficiency (evaluated as the ratio between the rectified DC power over the input RF power received by the patch antenna) of 30%, with 0 dBm of input power, as plotted in Figure 12b, which is comparable to the power conversion efficiencies that can be obtained for rectennas realized on conventional stiff RF substrates.

Regarding the inclusion of air gaps or air cavities in 3D-printed substrate material, with the intention of optimizing the gain and bandwidth of microstrip patch antennas, extensive work has been published [67,68]. In [69], a 2.4 GHz microstrip patch antenna was fabricated on a heterogeneous substrate composed of a dual extrusion fused deposition modeling printer consisting of acrylonitrile butadiene styrene (ABS) tiles surrounded by flexible NinjaFlex (NF). This peculiar combination of materials with similar electric permittivity allows for control of the RF performance, maintaining the substrate flexibility, ensuring performance stability with curvature, and increasing both the antenna bandwidth and gain up to 400% and 250%, respectively, by varying the ABS material percentage with respect to the NF.

Further investigations presented in the literature have the aim of optimizing the substrate design into a smart pattern of alternating air and 3D printable material that not only provides the required effective EM properties of the dielectric, offering satisfying device performance, but also the structure’s mechanical robustness to the inevitable external pressures and deformations caused by ordinary body movements. In this sense, the literature presents several studies about the optimization design of lightweight and robust structures for aerospace, naval, and industrial constructions, many of which are inspired by the internal biological structures of living organisms, such as animal bones, bamboo, beaks, and honeycombs [70,71,72]. In particular, honeycombs are widely recognized not only as lightweight and mechanically strong structures that are able to absorb external compressions and stretching but also as a good solution for minimizing insertion losses because of the low volume of the dielectric material, so they can therefore be exploited in many engineering fields [73,74].

**Figure 13:**
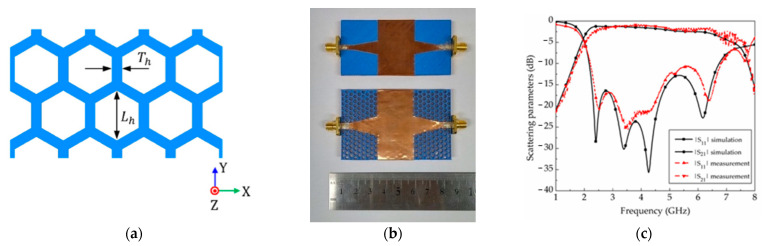
(**a**) Schematic of the proposed honeycomb structure with dimensions of the unit cells and thickness of the PLA spacing among them; (**b**) fabricated prototypes of the microstrip-fed SIW on solid (top) and honeycomb-shaped (bottom) PLA; (**c**) measured and simulated results of the considered SIWs [75].

An interesting application of 3D-printed honeycomb structures is presented in [75], where the authors propose a 3D-printed honeycomb-shaped SIW (Figure 13) using a lossy polylactic acid (PLA) filament with a dielectric constant ε_r_ and loss tangent tan (δ) of 2.2 and 0.05 at 3.5 GHz, respectively. Here, the focus is on the optimization of the honeycomb unit cells’ size, thickness, height, and spacing among them in order to reduce the average insertion losses of a microstrip-fed SIW. The implementation of these honeycomb substrates provided an average measured insertion loss of 1.38 dB compared to the 3.15 dB of an SIW realized on solid PLA, analyzed in the frequency range between 3.4 and 5.5 GHz. The achieved result is comparable to that of an SIW fabricated on commercial printed circuit boards (PCB), with the advantage of a 10% reduction in the weight and a 50% reduction in the printing time ensured by 3D printing.

## 5. Conclusions

This paper features an overview of some of the latest microwave solutions that have been adopted for wearable sensors and systems aimed at achieving the paradigms of IoT and e-Health. In particular, three different aspects have been analyzed that can be exploited for different e-Health applications and providing an examination of various innovative technologies. First, microwave wearable sensors were presented, focusing on microfluidic systems able to detect the concentration of aqueous solutions in contact with the body. Then, a portable microwave system with quasi-3D scanning capabilities was coupled with wearable electronics for indoor positioning and tracking, enabling remote fall alert capabilities. Finally, preliminary implementation of battery-less solutions, adopting additive manufacturing and 3D printing, were shown to demonstrate the possibility of exploiting non-conventional lossy and low-cost materials to realize pervasive devices and systems for wearable applications and integration in everyday objects.

Future perspectives about these kinds of wearable devices should preview the exploitation of the ever-increasing millimeter waves, not only to embrace the technological advancement directions but also to take advantage of the further miniaturization, and thus comfort, that can be achieved. Moreover, the full wearability of these sensors should be addressed, as well as their total energy autonomy, with the aim of removing the need for batteries and their related maintenance, paving the wave to the establishment of their future usage in the framework of any possible technological environment.

## Data Availability

Not applicable.

## References

[1] Roggen D., Magnenat S., Waibel M., Tröster G. (2011). Wearable Computing. IEEE Robot. Autom. Mag..

[2] Aroganam G., Manivannan N., Harrison D. (2019). Review on Wearable Technology Sensors Used in Consumer Sport Applications. Sensors.

[3] Vijayan V., Connolly J.P., Condell J., McKelvey N., Gardiner P. (2021). Review of Wearable Devices and Data Collection Considerations for Connected Health. Sensors.

[4] Castillejo P., Martinez J.-F., Rodriguez-Molina J., Cuerva A. (2013). Integration of wearable devices in a wireless sensor network for an E-health application. IEEE Wirel. Commun..

[5] Kim J., Chou E.-F., Le J., Wong S., Chu M., Khine M. (2019). Soft Wearable Pressure Sensors for Beat-to-Beat Blood Pressure Monitoring. Adv. Healthc. Mater..

[6] Kim Y., Jeung J., Song Y., Ko H., Park S., Park H., Jeon G., Chung Y. A Wearable System for Heart Rate Recovery Evaluation with Real-Time Classification on Exercise Condition. Proceedings of the 43rd Annual International Conference of the IEEE Engineering in Medicine & Biology Society (EMBC).

[7] Islam M., Manjur S.M. Design and Implementation of a Wearable System for Non-Invasive Glucose Level Monitoring. Proceedings of the IEEE International Conference on Biomedical Engineering, Computer and Information Technology for Health (BECITHCON).

[8] Cinel G., Tarim E.A., Tekin H.C. Wearable respiratory rate sensor technology for diagnosis of sleep apnea. Proceedings of the Medical Technologies Congress (TIPTEKNO).

[9] Brady S., Carson B., O’Gorman D., Moyna N., Diamond D. Combining wireless with wearable technology for the development of on-body networks. Proceedings of the International Workshop on Wearable and Implantable Body Sensor Networks (BSN’06).

[10] Newell D., Duffy M. (2019). Review of Power Conversion and Energy Management for Low-Power, Low-Voltage Energy Harvesting Powered Wireless Sensors. IEEE Trans. Power Electron..

[11] Chong Y.-W., Ismail W., Ko K., Lee C.-Y. (2019). Energy Harvesting for Wearable Devices: A Review. IEEE Sens. J..

[12] Khan A.S., Khan F.U. (2022). A survey of wearable energy harvesting systems. Int. J. Energy Res..

[13] Wang L., Fei Z., Qi Y., Zhang C., Zhao L., Jiang Z., Maeda R. (2022). Overview of Human Kinetic Energy Harvesting and Application. ACS Appl. Energy Mater..

[14] Padash M., Enz C., Carrara S. (2020). Microfluidics by Additive Manufacturing for Wearable Biosensors: A Review. Sensors.

[15] Huang J., Yu X., Wang Y., Xiao X. (2016). An Integrated Wireless Wearable Sensor System for Posture Recognition and Indoor Localization. Sensors.

[16] Lian H., Xue M., Ma K., Mo D., Wang L., Cui Z., Chen X. (2022). Three-Dimensional Printed Carbon Black/PDMS Composite Flexible Strain Sensor for Human Motion Monitoring. Micromachines.

[17] El Gharbi M., Fernández-García R., Ahyoud S., Gil I. (2020). A Review of Flexible Wearable Antenna Sensors: Design, Fabrication Methods, and Applications. Materials.

[18] Hasan M.N., Tamanna S., Singh P., Nadeem M.D., Rudramuni M. Cylindrical Dielectric Resonator Antenna Sensor for Non-Invasive Glucose Sensing Application. Proceedings of the 2019 6th International Conference on Signal Processing and Integrated Networks (SPIN).

[19] Wang L. (2018). Microwave Sensors for Breast Cancer Detection. Sensors.

[20] Schiavoni R., Monti G., Tedesco A., Tarricone L., Piuzzi E., de Benedetto E., Masciullo A., Cataldo A. Microwave Wearable System for Sensing Skin Hydration. Proceedings of the 2021 IEEE International Instrumentation and Measurement Technology Conference (I2MTC).

[21] Fletcher R.R., Kulkarni S. Clip-on wireless wearable microwave sensor for ambulatory cardiac monitoring. Proceedings of the 2010 Annual International Conference of the IEEE Engineering in Medicine and Biology.

[22] Mehrotra P., Chatterjee B., Sen S. (2019). EM-Wave Biosensors: A Review of RF, Microwave, mm-Wave and Optical Sensing. Sensors.

[23] Chen C.Y., Hsieh C.W., Liao Y.H., Yin T.J. Implementation of Wearable Devices and Indoor Positioning System for a Smart Hospital Environment. Proceedings of the 2018 International Symposium in Sensing and Instrumentation in IoT Era (ISSI).

[24] Pham M., Yang D., Sheng W. (2018). A Sensor Fusion Approach to Indoor Human Localization Based on Environmental and Wearable Sensors. IEEE Trans. Autom. Sci. Eng..

[25] Hirata Y., Komatsuda S., Kosuge K. Fall prevention control of passive intelligent walker based on human model. Proceedings of the International Conference on Intelligent Robots and Systems.

[26] Bet P., Castro P.C., Ponti M. (2019). Fall detection and fall risk assessment in older person using wearable sensors: A systematic review. Int. J. Med Inform..

[27] Chen J., Kwong K., Chang D., Luk J., Bajcsy R. Wearable Sensors for Reliable Fall Detection. Proceedings of the 2005 IEEE Engineering in Medicine and Biology 27th Annual Conference.

[28] Toda K., Shinomiya N. Fall Detection System for the Elderly Using RFID Tags with Sensing Capability. Proceedings of the 2018 IEEE 7th Global Conference on Consumer Electronics (GCCE).

[29] Shinmoto Torres L., Ranasinghe D.C., Shi Q., Sample A.P. Sensor enabled wearable RFID technology for mitigating the risk of falls near beds. Proceedings of the 2013 IEEE International Conference on RFID (RFID).

[30] Toda K., Shinomiya N. Machine learning-based fall detection system for the elderly using passive RFID sensor tags. Proceedings of the 2019 13th International Conference on Sensing Technology (ICST).

[31] Ahmed S., Tahir F.A., Shamim A., Cheema H.M. (2015). A Compact Kapton-Based Inkjet-Printed Multiband Antenna for Flexible Wireless Devices. IEEE Antennas Wirel. Propag. Lett..

[32] Aziz M.A., Roy S., Berge L.A., Irfanullah, Nariyal S., Braaten B.D. A conformal CPW folded slot antenna array printed on a Kapton substrate. Proceedings of the 2012 6th European Conference on Antennas and Propagation (EUCAP).

[33] Sabban A. Small New Wearable Antennas for IOT, Medical and Sport Applications. Proceedings of the 2019 13th European Conference on Antennas and Propagation (EuCAP).

[34] Zhang S., Njoku C.C., Whittow W.G., Vardaxoglou J.C. (2015). Novel 3D printed synthetic dielectric substrates. Microw. Opt. Technol. Lett..

[35] Escobedo P., Bhattacharjee M., Nikbakhtnasrabadi F., Dahiya R. (2021). Smart Bandage with Wireless Strain and Temperature Sensors and Batteryless NFC Tag. IEEE Internet Things J..

[36] Eldamak A.R., Fear E.C. (2018). Conformal and Disposable Antenna-Based Sensor for Non-Invasive Sweat Monitoring. Sensors.

[37] Mason A., Korostynska O., Louis J., Cordova-Lopez L.E., Abdullah B., Greene J., Connell R., Hopkins J. (2018). Noninvasive In-Situ Measurement of Blood Lactate Using Microwave Sensors. IEEE Trans. Biomed. Eng..

[38] Popovic Z., Momenroodaki P., Scheeler R. (2014). Toward wearable wireless thermometers for internal body temperature measurements. IEEE Commun. Mag..

[39] Khan A.N., Ermakov A., Sukhorukov G., Hao Y. (2019). Radio frequency controlled wireless drug delivery devices. Appl. Phys. Rev..

[40] Baghelani M., Abbasi Z., Daneshmand M., Light P.E. (2020). Non-invasive continuous-time glucose monitoring system using a chipless printable sensor based on split ring microwave resonators. Sci. Rep..

[41] Mertens M., Chavoshi M., Peytral-Rieu O., Grenier K., Schreurs D. (2023). Dielectric Spectroscopy: Revealing the True Colors of Biological Matter. IEEE Microw. Mag..

[42] Ebrahimi A., Withayachumnankul W., Al-Sarawi S.F., Abbott D. Microwave microfluidic sensor for determination of glucose concentration in water. Proceedings of the 2015 IEEE 15th Mediterranean Microwave Symposium (MMS).

[43] McCaul M., Porter A., Barrett R., Wallace G., Stroiescu F., Wallace G.G., Diamond D. (2018). Wearable Platform for Real-time Monitoring of Sodium in Sweat. Chem. Phys. Chem..

[44] Chen T., Dubuc D., Poupot M., Fournie J.-J., Grenier K. (2012). Accurate Nanoliter Liquid Characterization Up to 40 GHz for Biomedical Applications: Toward Noninvasive Living Cells Monitoring. IEEE Trans. Microw. Theory Techn..

[45] Rose D.P., Ratterman M.E., Griffin D.K., Hou L., Kelley-Loughnane N., Naik R.R., Hagen J.A., Papautsky I., Heikenfeld J.C. (2015). Adhesive RFID Sensor Patch for Monitoring of Sweat Electrolytes. IEEE Trans. Biomed. Eng..

[46] Benassi F., Paolini G., Masotti D., Costanzo A. (2021). A Wearable Flexible Energy-Autonomous Filtenna for Ethanol Detection at 2.45 GHz. IEEE Trans. Microw. Theory Techn..

[47] Su W., Cook B.S., Tentzeris M.M. (2016). Additively Manufactured Microfluidics-Based “Peel-and-Replace” RF Sensors for Wearable Applications. IEEE Trans. Microw. Theory Techn..

[48] Artis F., Chen T., Chretiennot T., Fournie J.-J., Poupot M., Dubuc D., Grenier K. (2015). Microwaving biological cells: Intracellular analysis with microwave dielectric spectroscopy. IEEE Microw. Mag..

[49] Salim A., Kim S.-H., Park J.Y., Lim S. (2018). Microfluidic Biosensor Based on Microwave Substrate-Integrated Waveguide Cavity Resonator. J. Sens..

[50] Noda A., Shinoda H. On-body sensor node localization using reference RFID tags embedded in wearable waveguide. Proceedings of the 2016 IEEE Sensors.

[51] Liu Z., Fu Z., Li T., White I.H., Penty R.V., Yang X., Du R., Crisp M. (2022). A Phase and RSSI-Based Method for Indoor Localization Using Passive RFID System with Mobile Platform. IEEE J. Radio Freq. Identif..

[52] Amendola S., Bianchi L., Marrocco G. (2015). Movement Detection of Human Body Segments: Passive radio-frequency identification and machine-learning technologies. IEEE Antennas Propag. Mag..

[53] Islam S.M.M., Lubecke V.M. (2022). Sleep Posture Recognition with a Dual-Frequency Microwave Doppler Radar and Machine Learning Classifiers. IEEE Sens. Lett..

[54] Zhang H., Zhang Z., Gao N., Xiao Y., Meng Z., Li Z. (2020). Cost-Effective Wearable Indoor Localization and Motion Analysis via the Integration of UWB and IMU. Sensors.

[55] Abdi A., Kaveh M. (2011). A Comparative Study of Two Shadow Fading Models in Ultrawideband and Other Wireless Systems. IEEE Trans. Wirel. Commun..

[56] Hongmei Z., Hailong Y., Shuting G. (2016). Research on path loss and shadow fading of ultra wideband simulation channel. Int. J. Distrib. Sens. Netw..

[57] Paolini G., Masotti D., Antoniazzi F., Cinotti T.S., Costanzo A. (2019). Fall Detection and 3-D Indoor Localization by a Custom RFID Reader Embedded in a Smart e-Health Platform. IEEE Trans. Microw. Theory Tech..

[58] Paolini G., Masotti D., Costanzo A. RFID Reader and Wearable Tags for Smart Health Applications. Proceedings of the 2021 XXXIVth General Assembly and Scientific Symposium of the International Union of Radio Science (URSI GASS).

[59] Waheed M., Afzal H., Mehmood K. (2021). NT-FDS—A Noise Tolerant Fall Detection System Using Deep Learning on Wearable Devices. Sensors.

[60] Wang Y., Wu K., Ni L.M. (2017). WiFall: Device-Free Fall Detection by Wireless Networks. IEEE Trans. Mob. Comput..

[61] Li M., Yang Y. (2023). Single- and Multiple-Material Additively Manufactured Electronics: A Further Step from the Microwave-to-Terahertz Regimes. IEEE Microw. Mag..

[62] Battistini G., Paolini G., Masotti D., Costanzo A. Innovative 3-D Printing Processing Techniques for Flexible and Wearable Planar Rectennas. Proceedings of the 2022 Wireless Power Week (WPW).

[63] Latti K.-P., Kettunen M., Strom J.-P., Silventoinen P. (2007). A Review of Microstrip T-Resonator Method in Determining the Dielectric Properties of Printed Circuit Board Materials. IEEE Trans. Instrum. Meas..

[64] Herrick K., Schwarz T., Katehi L. (1998). Si-micromachined coplanar waveguides for use in high-frequency circuits. IEEE Trans. Microw. Theory Tech..

[65] Battistini G., Paolini G., Masotti D., Costanzo A. Wearable Coplanar-Fed 2.45 GHz-Rectenna on a Flexible 3D-Printable Low-Cost Substrate. Proceedings of the 2022 52nd European Microwave Conference (EuMC).

[66] Papapolymerou I., Franklin Drayton R., Katehi L.P.B. (1998). Micromachined patch antennas. IEEE Trans. Antennas Propag..

[67] Baines W., Dahle R. Enhanced bandwidth microstrip patch antennas through 3-D printing. Proceedings of the 2016 IEEE International Symposium on Antennas and Propagation (APSURSI).

[68] Moscato S., Bahr R., Le T., Pasian M., Bozzi M., Perregrini L., Tentzeris M.M. (2016). Infill-Dependent 3-D-Printed Material Based on NinjaFlex Filament for Antenna Applications. IEEE Antennas Wirel. Propag. Lett..

[69] Ramadan M., Dahle R. (2019). Characterization of 3-D Printed Flexible Heterogeneous Substrate Designs for Wearable Antennas. IEEE Trans. Antennas Propag..

[70] Zhang Q., Yang X., Li P., Huang G., Feng S., Shen C., Han B., Zhang X., Jin F., Xu F. (2015). Bioinspired engineering of honeycomb structure—Using nature to inspire human innovation. Prog. Mater. Sci..

[71] Zou M., Xu S., Wei C., Wang H., Liu Z. (2016). A bionic method for the crashworthiness design of thin-walled structures inspired by bamboo. Thin Walled Struct..

[72] Hribar K.C., Soman P., Warner J., Chung P., Chen S. (2014). Light-assisted direct-write of 3D functional biomaterials. Lab Chip.

[73] Yap Y.L., Yeong W.Y. (2015). Shape recovery effect of 3D printed polymeric honeycomb. Virtual Phys. Prototyp..

[74] Ijaz H., Saleem W., Zain-ul-Abdein M., Mabrouki T., Rubaiee S., Salmeen Bin Mahfouz A. (2017). Finite Element Analysis of Bend Test of Sandwich Structures Using Strain Energy Based Homogenization Method. Adv. Mater. Sci. Eng..

[75] Kim Y., Tentzeris M.M., Lim S. (2019). Low-Loss and Light Substrate Integrated Waveguide Using 3D Printed Honeycomb Structure. Materials.

